# Benchmarking the performance of large language models on medical genetics questions: a comparative evaluation of GPT-5.1, Gemini Pro 3, and Claude Opus 4.5

**DOI:** 10.3389/fmed.2026.1946188

**Published:** 2026-10-13

**Authors:** Muserref Basdemirci, Melek Tassoker

**Affiliations:** 1 Department of Medical Genetics, Konya City Hospital, Konya, Türkiye; 2 Department of Dentomaxillofacial Radiology, Faculty of Dentistry, Necmettin Erbakan University, Konya, Türkiye

**Keywords:** artificial intelligence, benchmarking, chatGPT, genetics, medical, language models

## Abstract

**Objective:**

This study benchmarks the performance of three state of the art large reasoning models comprising GPT 5.1, Gemini Pro 3, and Claude Opus 4.5 by evaluating their accuracy, response consistency, processing speed, and output length on medical genetics questions from the Turkish Medical Specialty Examination (TUS).

**Methods:**

A total of 233 medical genetics questions were selected based on predefined inclusion criteria covering molecular mechanisms, inheritance patterns, genetic disorders, and diagnostic techniques. Each question was independently submitted to GPT 5.1, Gemini Pro 3, and Claude Opus 4.5 using a standardized prompt. Model accuracy was compared using McNemar's test. Response consistency was assessed using Cohen's Kappa. Response time and word count were analyzed using the Friedman test with Dunn Bonferroni *post hoc* comparisons.

**Results:**

No statistically significant differences were observed in overall accuracy among the models (*p* > 0.05). GPT 5.1 achieved the highest accuracy at 97.9 percent followed by Claude Opus 4.5 at 96.6 percent and Gemini Pro 3 at 96.1 percent. Cohen's Kappa indicated moderate qualitative divergence between Gemini Pro 3 and Claude Opus 4.5 with kappa equal to 0.451. Significant differences were found in response time and output length (*p* < 0.001).

**Conclusion:**

Although all models demonstrate similarly high accuracy, they differ in operational behavior, suggesting distinct internal knowledge representations and response strategies.

## Introduction

In recent years, large language models (LLMs) have rapidly evolved into large reasoning models, enabling multi-step planning, autonomous agent-like behaviors, and multimodal information synthesis. As the leading representatives of this technological leap, OpenAI's GPT-5.1, Google DeepMind's Gemini Pro 3, and Anthropic's Claude Opus 4.5 offer solutions to complex data-intensive problems through distinct architectural philosophies and operational capabilities (1–3).

Evaluation and assessment are fundamental components of medical education. The Turkish Medical Specialty Examination (TUS) is a national standardized exam taken after completion of a six-year medical degree and plays a central role in the professional advancement of physicians in Türkiye by determining placement into specialty training programs. The exam consists of two main sections, clinical sciences and basic sciences, each comprising 120 multiple-choice questions (4).

LLMs have been trained on massive datasets from the medical literature, enabling them to answer complex medical questions. Recent studies have reported high accuracy in solving complex cases and answering examination questions across various medical disciplines, including radiology, anatomy, and pathology (5–9) Medical genetics encompasses a broad range of knowledge and reasoning tasks, including molecular mechanisms, inheritance patterns, cytogenetic abnormalities, genetic diagnosis, and clinical interpretation, providing a relevant context for assessing LLM performance across diverse genetics-related questions. TUS genetics questions assess a wide range of competencies, including molecular mechanisms, cytogenetic disorders, inheritance patterns, and genetic counseling. To date, no study has systematically evaluated LLM performance on TUS-derived medical genetics questions. Therefore, this study aimed to benchmark GPT-5.1, Gemini Pro 3, and Claude Opus 4.5 in terms of accuracy, response time, and response length using a standardized question set.

## Methods

### Study design

This benchmarking study evaluated the performance of three LLMs—GPT-5.1 (OpenAI), Gemini Pro 3 (Google DeepMind), and Claude Opus 4.5 (Anthropic)—on medical genetics questions from the Turkish Medical Specialty Examination (TUS). Primary outcomes were accuracy, response time, and response length (word count).

### Question selection and dataset

A total of 233 medical genetics questions from TUS examinations conducted between 2011 and 2025 were included (Suppl File). Questions were manually selected according to predefined criteria. As the TUS does not contain a dedicated medical genetics section, genetics-related questions are distributed across the basic and clinical sciences sections. Therefore, an operational criterion was used to standardize the identification of genetics-related questions and reduce subjective selection. Eligible questions involved DNA, RNA, chromosomes, genes, mutations, inheritance, genetic diseases or syndromes, genetic diagnostic tests, or prenatal diagnosis. Questions were included only if more than two answer choices contained genetic diseases or syndromes. This operational criterion was applied to provide a consistent and reproducible approach to question selection; however, it may have excluded some questions assessing genetic concepts in which the answer choices were not primarily disease- or syndrome-based. Questions lacking clinical genetic relevance were excluded.

### Model access and prompting

All models were accessed through their official web-based platforms during the same evaluation period. Each question was submitted in a separate new session to avoid contextual memory effects. The following standardized prompt was used for all models: “*You are a medical genetics expert. Select the single best answer and briefly justify your choice.”* No additional instructions, plugins, or external tools were used.

### Outcome measures

Accuracy was defined as agreement with the official TUS answer key. Response time was measured in seconds from question submission to completion of the final response using a digital stopwatch. Response length was calculated as the total word count of each response, including the justification.

### Evaluation procedure

For each model response, answer correctness, response time, and word count were recorded in a spreadsheet for subsequent analysis. Answer correctness was determined by comparison with the official TUS answer key, which served as the gold standard. This evaluation was performed by a single reviewer experienced in medical genetics.

### Additional analysis using newly generated questions

To assess the potential influence of prior exposure to publicly available TUS questions, an additional set of 40 medical genetics multiple-choice questions was developed by MB, a medical genetics specialist. The questions were newly constructed and were not derived from or adapted from previously published TUS examinations. The correct answers were determined based on established medical genetics textbooks. The questions were administered to all three models using the same standardized prompting procedure as the primary dataset.

### Statistical analysis

Statistical analyses were performed using SPSS version 21.0 (IBM, Armonk, NY), with *p* < 0.05 considered statistically significant. Cochran's *Q* test and McNemar's test were used to compare paired accuracy rates between models. Cohen's Kappa analysis assessed agreement and similarity of error patterns. Because response times and word counts were not normally distributed, Friedman tests were used for overall comparisons, followed by Dunn–Bonferroni *post hoc* tests with Bonferroni correction for pairwise analyses. In addition, a *post hoc* content-based review was conducted for all questions answered incorrectly by at least one model to explore whether errors clustered within particular genetics-related topics. Each incorrectly answered question was classified according to its primary content area.

### Ethical considerations

The study used publicly available examination questions and did not involve human participants or identifiable personal data; therefore, ethical approval was not required.

## Results

The total number of true and false answers and the accuracy rates for LLMs are presented in Table 1. McNemar's test revealed no statistically significant differences in accuracy levels between the models. The comparison between ChatGPT-5.1 and Gemini-3Pro was not significant (*p* = 0.219), nor was the comparison between ChatGPT-5 and Claude-4.5 (*p* = 0.375). Similarly, no significant difference was observed between Gemini-3Pro and Claude-4.5 (*p* = 1.000). All comparisons were performed on 233 questions.

**Table 1:** Accuracy rates and performance distribution of LLM models.

Model	True	False	Total	Accuracy (%)
ChatGPT-5.1	228	5	233	97.9%
Claude-4.5	225	8	233	96.6%
Gemini-3Pro	224	9	233	96.1%

Furthermore, Cohen's Kappa analyses, which measure the consistency between the models’ responses, revealed that their knowledge bases and error patterns diverge significantly (Table 2). These findings demonstrate that although the models exhibit similar overall success rates, they diverge qualitatively, with Gemini-3Pro and Claude-4.5, in particular, showing a tendency to fail on different types of questions.

**Table 2:** Comparative agreement analysis (Cohen's kappa).

Model comparison	Kappa (*κ*) value	*p*-value	Strength of agreement
ChatGPT-5.1 & Claude-4.5	.605	<.001	Moderate to Substantial
ChatGPT-5.1 & Gemini-3Pro	.559	<.001	Moderate
Gemini-3Pro & Claude-4.5	.451	<.001	Moderate

Descriptive analysis of response times demonstrated that Gemini-3Pro had the shortest mean response time (6.69 ± 1.31 s), followed by ChatGPT-5.1 (8.41 ± 2.72 s), while Claude-4.5 exhibited the longest response time (16.62 ± 2.71 s). The mean rank values also indicated that Gemini-3Pro was the fastest model and Claude-4.5 was the slowest.

Pairwise comparisons revealed statistically significant differences in response times between all model pairs (all *p* < .001). Gemini-3Pro responded significantly faster than both ChatGPT-5.1 and Claude-4.5, while ChatGPT-5 also demonstrated significantly shorter response times compared to Claude-4.5.

Descriptive analysis of word counts showed that ChatGPT-5.1 generated the shortest responses, with a mean of 89.35 ± 34.69 words, whereas Claude-4.5 produced moderately longer responses (138.08 ± 27.17 words). Gemini-3Pro generated the longest responses overall, with a mean word count of 244.09 ± 80.54 words. Mean rank values similarly indicated that Gemini-3Pro produced the highest number of words, while ChatGPT-5.1 generated the fewest (Table 3). Pairwise comparisons demonstrated statistically significant differences in word counts between all model pairs (all *p* < .001). Gemini-3Pro produced significantly longer responses than both ChatGPT-5.1 and Claude-4.5, while Claude-4.5 also generated significantly more words than ChatGPT-5.

**Table 3:** Descriptive statistics of word count across models.

Model	Mean (Words)	Std. deviation	Min	Max	Mean rank
ChatGPT-5.1	89.35	34.69	18	207	1.07
Claude-4.5	138.08	27.17	61	205	1.97
Gemini-3Pro	244.09	80.54	78	649	2.96

A *post hoc* multi-model consensus analysis using the question-level responses of the three models showed that the 2-of-3 majority-vote approach, in which the consensus was considered correct when at least two models provided the correct answer, achieved an overall accuracy of 98.3% (229/233), slightly exceeding the accuracy of the best-performing individual model (97.9%) and demonstrating complementary error patterns among the models (Table 4).

**Table 4:** Question-level response patterns and multi-model consensus performance.

Response pattern	GPT-5.1	Gemini Pro 3	Claude Opus 4.5	*n* (%)	Majority vote
All models correct	Correct	Correct	Correct	219 (94.0)	Correct
Gemini Pro 3 only incorrect	Correct	Incorrect	Correct	5 (2.1)	Correct
Claude Opus 4.5 only incorrect	Correct	Correct	Incorrect	4 (1.7)	Correct
GPT-5.1 only incorrect	Incorrect	Correct	Correct	1 (0.4)	Correct
All models incorrect	Incorrect	Incorrect	Incorrect	4 (1.7)	Incorrect
Overall accuracy	97.9% (228/233)	96.1% (224/233)	96.6% (225/233)	233 (100)	98.3% (229/233)

A *post hoc* content-based review of the 14 questions answered incorrectly by at least one model revealed heterogeneous error topics, with no clear concentration in a specific genetics-related content area (Table 5).

**Table 5:** Content-based analysis of questions answered incorrectly by at least one large language model.

Study question no.	Question topic	Content category	GPT-5.1	Gemini Pro 3	Claude Opus 4.5
21	Purine ring structure	Basic molecular/biochemical genetics	Correct	Correct	Incorrect
44	Fetal alcohol syndrome and phenotypic recognition	Clinical syndrome recognition	Correct	Correct	Incorrect
61	Polycythemia vera and associated findings	Hematologic/cancer genetics	Incorrect	Incorrect	Incorrect
74	Hamartomatous polyposis syndromes	Hereditary cancer syndromes	Incorrect	Correct	Correct
94	Differential diagnosis of thrombocytosis (JAK2, t(9;22))	Hematologic/molecular diagnostics	Correct	Correct	Incorrect
96	Clinical diagnosis of Maroteaux–Lamy syndrome	Metabolic/genetic syndromes	Correct	Incorrect	Correct
101	46,XY gonadal dysgenesis and internal genital development	Reproductive/endocrine genetics	Correct	Incorrect	Correct
144	Ultrasonographic findings associated with Down syndrome	Prenatal/chromosomal genetics	Correct	Correct	Incorrect
152	Mitral valve prolapse and hereditary disorders	Connective tissue/genetic syndromes	Correct	Incorrect	Correct
162	Diagnostic criteria for celiac disease	Immunogenetics/clinical diagnosis	Incorrect	Incorrect	Incorrect
166	Genes involved in pancreatic adenocarcinoma	Cancer genetics	Incorrect	Incorrect	Incorrect
186	Congenital adrenal hyperplasia in a 46,XY infant	Reproductive/endocrine genetics	Correct	Incorrect	Correct
198	Leukocyte adhesion deficiency type I	Primary immunodeficiency/immunogenetics	Correct	Incorrect	Correct
222	Nucleotide-to-protein length calculation	Basic molecular genetics	Incorrect	Incorrect	Incorrect

To address the potential influence of training data contamination, the models were additionally evaluated using 40 newly generated medical genetics questions that were not derived from previously published TUS examinations. GPT correctly answered all 40 questions (100.0%), followed by Claude with 39 correct responses (97.5%) and Gemini with 38 correct responses (95.0%). No statistically significant difference in accuracy was observed among the three models (Cochran's Q = 3.00, df = 2, *p* = 0.223). Pairwise exact McNemar tests likewise revealed no significant differences between GPT and Gemini (*p* = 0.500), GPT and Claude (*p* = 1.000), or Gemini and Claude (*p* = 1.000).

## Discussion

The field of medicine is increasingly shaped by artificial intelligence, with LLMs representing one of its most rapidly evolving components. This study benchmarks GPT-5.1, Gemini Pro 3, and Claude Opus 4.5 using medical genetics questions from the Turkish Medical Specialty Examination, which includes both knowledge-based and case-based items requiring interpretation and analysis. This examination setting reflects a broader clinical need, as the global shortage of genetics specialists contrasts with the large number of rare genetic disorders. Given the complexity and multisystem nature of these conditions, early diagnosis is essential, yet patients often experience long diagnostic delays, particularly in resource-limited settings. In this context, the high accuracy and rapid information synthesis capabilities of LLMs may offer significant potential to support genetic diagnosis and mitigate workforce shortages (10–13).

The findings of this study demonstrate that current LLMs achieve extremely high and closely comparable accuracy rates in answering complex questions in the field of medical genetics (96.1%–97.9%). For contextual reference, published statistics from the 2020 s-term TUS reported mean correct-response rates of 44.11% for the Basic Medical Sciences Test and 54.81% for the Clinical Medical Sciences Test (https://medimagazin.com.tr/guncel/osym-degerlendirme-raporunu-yayimladi-iste-tusta-en-zor-brans-93723)*.* However, these aggregate examination-level statistics are not specific to medical genetics and therefore cannot be directly compared with the LLM performance observed in the present study. The absence of a statistically significant difference in performance between the models (*p* > 0.05) suggests that current LLMs demonstrate similarly high levels of performance for the types of medical genetics questions evaluated in this study. However, this finding should not be generalized to more complex or challenging genetics questions, for which greater differences in model performance may emerge. Furthermore, this similarity in overall accuracy does not imply that the models operate in a qualitatively identical manner. The results of Cohen's Kappa analysis reveal only a *moderate* level of agreement between the models, highlighting a critical insight: although the models obtain similar scores, the specific questions they answer incorrectly and the underlying knowledge structures they rely on differ substantially. In particular, the low agreement observed between Gemini-3 Pro and Claude-4.5 suggests that these models exhibit distinct error patterns across different question types and clinical scenarios. The *post hoc* multi-model consensus analysis further showed that a 2-of-3 majority-vote approach achieved an accuracy of 98.3% (229/233), slightly exceeding the accuracy of the best-performing individual model (GPT-5.1, 97.9%). Although the magnitude of improvement was modest, this finding suggests that combining complementary model outputs may have the potential to improve reliability. However, this *post hoc* majority-vote analysis does not represent direct cross-validation between models, as the models did not evaluate each other's outputs. Prospective studies involving explicit model-to-model cross-checking are needed to determine whether such strategies provide a meaningful advantage in clinical decision support. From an operational perspective, significant differences in response time and response length were observed among the models. Gemini-3 Pro was the fastest model (mean 6.69 s) while producing the longest responses (mean 244 words). ChatGPT-5 generated the most concise responses (mean 89 words), whereas Claude-4.5 showed the longest response time (mean 16.62 s) and produced moderately longer responses than ChatGPT-5 (mean 138 words). Response length, however, is inherently prompt-dependent, and more restrictive instructions regarding response format or word count could substantially reduce the observed differences between models. Thus, these findings should be interpreted as reflecting model behavior under the standardized prompt used in this study rather than as fixed model-specific characteristics. Similarly, the practical relevance of response-time differences of several seconds for individual interactions in a chat-based interface remains uncertain. Response time may also be influenced by external factors such as internet connectivity, platform variability, and system load. Accordingly, the observed response-time differences should be interpreted cautiously and should not be considered indicative of clinically meaningful performance differences.

The high performance observed in the present study can also be considered within the broader context of AI evaluation across other medical specialties. In obstetrics and gynecology, Ługowski et al. (14) evaluated ChatGPT-3.5 and ChatGPT-4 using 352 questions from the Polish Specialty Certificate Examination and reported that ChatGPT-4 achieved accuracy rates of 83.2–89.6% when questions were presented in English, with performance also varying according to question difficulty. These findings, together with the higher accuracy observed in our medical genetics dataset, highlight the substantial knowledge capabilities of contemporary LLMs while also demonstrating that benchmark performance may vary according to model generation, specialty, and question characteristics. Importantly, the role of AI in medicine is increasingly extending beyond text-based examination tasks toward clinically oriented applications integrating heterogeneous data sources. A recent review of AI in fetal growth disorders (15) described applications combining maternal and fetal characteristics, biomarkers, Doppler indices, and ultrasound imaging, including deep learning–based automated fetal biometry and image analysis. This progression from knowledge-based benchmarking toward multimodal diagnostic applications underscores the need to evaluate AI systems not only for examination accuracy but also for their reliability and clinical validity in real-world settings.

Although there are currently no published studies in the literature evaluating GPT-5.1, Claude 4.5, and Gemini Pro 3 specifically in the field of medical genetics, existing research indicates that GPT-4 achieves a relatively high diagnostic accuracy of approximately 89%–90% when identifying genetic diseases from textbook-style clinical descriptions. However, in scenarios where real individuals describe their own symptoms and medical histories, the reported accuracy drops substantially to around 20%–21% (16). GeneT (17), a customized model for filtering rare disease variants, reduced the manual analysis time of experts from 60 min to 3 min, achieving a 20-fold increase in efficiency. GP-GPT outperformed GPT-4 and standard LLaMA models in tasks related to gene–phenotype matching and genomic relationship inference (18). Although LLMs have the potential to revolutionize the diagnosis of rare genetic diseases, phenotype extraction, variant interpretation, rare disorder management, genetic counseling support, and genomic data analysis, the current literature highlights both their transformative advantages and critical limitations in terms of reliability (17–23). Although it demonstrated strong performance on medical examinations such as the USMLE, similar to its success on the TUS questions in this study, it has been reported that the model may hallucinate non-existent gene symbols or show a bias toward frequently cited genes in the literature (e.g., *BRCA1, TP53, PTEN*). Therefore, it is concluded that it has not yet reached a level at which it can replace expert-level genomic analysis tools (22).

## Limitations

This study has several limitations. First, the primary evaluation was based on multiple-choice questions from a standardized national examination, which predominantly assess recognition-based knowledge and do not fully capture real clinical reasoning, uncertainty, or contextual decision-making. Second, the lack of a human comparison group limits interpretation of performance, as comparable results might be achieved by medical students or residents. Future studies should directly compare LLM performance with medical students, residents, or medical genetics experts using the same question set under standardized conditions. Third, although the models were prompted to briefly justify their answers, the quality and factual accuracy of these explanations were not qualitatively assessed, as the predefined outcomes focused on answer accuracy, response time, and response length. Future studies should incorporate expert- and non-expert-based assessments of explanation quality. Additionally, each question was evaluated only once in an independent session; therefore, the reproducibility and stability of model responses across repeated queries could not be assessed. Future studies should incorporate repeated independent queries within the same evaluation period to assess response stability. Finally, the near-ceiling accuracy observed across all three models may have limited the ability of the benchmark to discriminate between model performance. Moreover, as all TUS questions included in the primary analysis were publicly available, their potential inclusion in the training data of the evaluated LLMs cannot be excluded. To partially address this concern, we conducted an additional analysis using 40 newly generated medical genetics questions that were not derived from or adapted from previously published TUS examinations. Although the models maintained high accuracy on this new question set, complete exclusion of overlap with information encountered during model training cannot be independently verified. Therefore, potential training data contamination remains a limitation, and the observed high performance should not be interpreted as evidence of true clinical reasoning.

## Conclusions

This study demonstrates that GPT-5.1, Gemini Pro 3, and Claude Opus 4.5 achieve similarly high accuracy on medical genetics questions from the Turkish Medical Specialty Examination. However, the models differ significantly in response time, output length, and error patterns. These results suggest that LLM evaluation should extend beyond accuracy to include operational and qualitative metrics. While these models show strong potential in medical education, they should be considered supportive tools rather than replacements for human clinical reasoning.

## Data Availability

The Turkish Medical Specialty Examination (TUS) questions and official answer keys used as source material in this study are publicly available through the official website of the Turkish Measurement, Selection and Placement Center (ÖSYM): https://www.osym.gov.tr/SinavGrubu/Index/6?yil=2010. The study included 233 medical genetics questions selected from TUS examinations administered between 2011 and 2025. The study-generated dataset used for the analyses is available from the corresponding author upon reasonable request.
